# Climatic oscillations in Quaternary have shaped the co-evolutionary patterns between the Norway spruce and its host-associated herbivore

**DOI:** 10.1038/s41598-020-73272-0

**Published:** 2020-10-05

**Authors:** Jakub Goczał, Andrzej Oleksa, Robert Rossa, Igor Chybicki, Katarzyna Meyza, Radosław Plewa, Matti Landvik, Mauro Gobbi, Gernot Hoch, Vytautas Tamutis, Maksims Balalaikins, Dmitry Telnov, Maria-Magdalena Dascălu, Adam Tofilski

**Affiliations:** 1 Department of Forest Ecosystems Protection, Faculty of Forestry, University of Agriculture in Krakow, 29 Listopada 46, 31-425 Kraków, Poland; 2 Department of Genetics, Faculty of Biological Sciences, Kazimierz Wielki University, Powstańców Wielkopolskich 10, 85-090 Bydgoszcz, Poland; 3 Department of Forest Protection, Forest Research Institute, Sękocin Stary, Sękocin Stary, Braci Leśnej 3, 05-090 Raszyn, Poland; 4 Tainionkoskentie 26 a. 2, 55100 Imatra, Finland; 5 Section of Invertebrate Zoology and Hydrobiology, MUSE-Science Museum, Corso del Lavoro e della Scienza 3, 38122 Trento, Italy; 6 BFW – Austrian Research Centre for Forests, Seckendorff-Gudent-Weg 8, 1131 Vienna, Austria; 7 Kaunas Botanical Garden, Vytautas Magnus University, Ž.E. Žilibero Str. 6, 46324 Kaunas, Lithuania; 8 Institute of Life Sciences and Technology, Daugavpils University, Vienibas 13, Daugavpils, 5400 Latvia; 9 Department of Life Sciences, Natural History Museum, London, SW7 5BD UK; 10 Institute of Biology, University of Latvia, Miera iela 3, Salaspils, Latvia; 11 Research Group in Invertebrate Diversity and Phylogenetics, Faculty of Biology, Alexandru Ioan Cuza University, Bd. Carol I, nr. 11, 700506 Iasi, Romania; 12 Department of Zoology and Animal Welfare, University of Agriculture in Krakow, Adama Mickiewicza 24/28, 30-059 Kraków, Poland

**Keywords:** Biogeography, Entomology, Coevolution, Speciation, Taxonomy

## Abstract

During the Last Glacial Maximum in the Northern Hemisphere, expanding ice sheets forced a large number of plants, including trees, to retreat from their primary distribution areas. Many host-associated herbivores migrated along with their host plants. Long-lasting geographic isolation between glacial refugia could have been led to the allopatric speciation in separated populations. Here, we have studied whether the migration history of the Norway spruce *Picea abies* in Quaternary has affected its host-associated herbivorous beetle—*Monochamus sartor*. By using microsatellite markers accompanied by the geometric morphometrics analysis of wing venation, we have revealed the clear geographic structure of *M. sartor* in Eurasia, encompassing two main clusters: southern (Alpine–Carpathian) and eastern (including northeastern Europe and Asia), which reflects the northern and southern ecotypes of its host. The two beetles’ lineages probably diverged during the Pleniglacial (57,000—15,000 BC) when their host tree species was undergoing significant range fragmentation and experienced secondary contact during post-glacial recolonization of spruce in the Holocene. A secondary contact of divergent lineages of *M.* sartor has resulted in the formation of the hybrid zone in northeastern Europe. Our findings suggest that the climatic oscillations during the Pleistocene have driven an insect-plant co-evolutionary process, and have contributed to the formation of the unique biodiversity of Europe.

## Introduction

The climatic fluctuations over the past two million years have had a pronounced impact on the distribution and evolution of temperate biota. Moving glaciers have forced many plants and animals to retreat from their primary distribution areas. When the glaciers retreated, the species started the recolonization of the previously inhospitable areas. In many taxa, the long–lasting geographic isolation that prevented or limited the gene flow resulted in a deep genetic divergence of isolated populations which might have finally led to the formation of new species^1–8^.

Changes in the distribution ranges of many plant species caused by the Quaternary climatic oscillations also had profound implications for other associated organisms, including herbivorous fauna. This co-dependence might be particularly noticeable in the case of woody plants, which constituted hosts for a large number of herbivorous insects. The Norway spruce (*Picea abies* (L.) H. Karst) may serve as an excellent example. This tree species is the host for almost four hundred different invertebrates^9,10^. Some of them are obligatorily associated with spruce, and therefore their evolutionary history is supposed to be tightly associated with the history of their host plant.

The phylogeography of Norway spruce is well-studied, and supported by both molecular and pollen data^11–15^. The Norway spruce in Europe has a disjunctive range, shaped by the Pleistocene climatic oscillations. The northern range encompassing northeastern Poland, northern Belarus, the Baltic countries, Fennoscandia, and the northern part of European Russia was found to be colonized from a single refugium located in the Russian plains^12,15^. In contrast, the southern spruce range running from southern Poland through the Carpathians and the Alps originated from several smaller and sparse mountain refugia located within the Alps and the Carpathian mountain chains^11,12,16^. What is particularly interesting, the Białowieża Forest—a large forest complex located in northeastern Poland—seems to be the most likely zone where the two spruce ranges had met and hybridized during the secondary contact, probably in the Atlantic chronozone (8,000–5,000 BP)^14^. Nevertheless, northern and southern spruce ranges are currently separated by a “spruceless zone” running through the Middle–Polish Plains^12,14^. The two ecotypes exhibit deep genetic divergence^11,14^, and also differ morphologically and phenologically^17^.

The Norway spruce is, therefore, an excellent model to study how past range fluctuations have affected the evolution of its host–associated herbivores. So far, only a few studies aimed at exploring this issue have been conducted on bark beetles^18–22^. However, conclusions from these studies are inconsistent. The oldest study based on isozyme analysis and the COI mitochondrial marker suggested a significant effect of the Norway spruce glacial history on the genetic structure of *Ips typographus* (Linnaeus, 1758)^19^, whereas similar research based on nuclear microsatellite markers showed the homogenous genetic structure of *I. typographus* in Europe^18^. In contrast, recent comprehensive studies based on a multilocus genetic approach provided strong evidence for the occurrence of genetically distinct groups in *I. typographus* and *Pityogenes chalcographus* (Linnaeus, 1760) corresponding to the northern and southern spruce ranges^21,22^.

It should be noted that in some cases, patterns of genetic variation may be more influenced by the species’ life–history traits, rather than by past migration routes^21^. This may be particularly true for bark beetles (Scolytidae), which are characterized by their considerable dispersal abilities and high demographic fluctuations. Bark beetles are relatively small insects that can be carried by the wind over tens of kilometres^23^, and the crossbreeding of individuals from distant populations may overwrite original patterns of variation in a short time^21^. Moreover, the rapid population declines following outbreaks may have led to the loss of rare alleles, which may have ultimately resulted in low genetic diversity^18^.

For several reasons, sawyers *Monochamus* Dejean, 1821 (Coleoptera: Cerambycidae) seem to be more suitable model organisms for comparative phylogeographic studies than bark beetles. They are too heavy for wind dispersion, and thus, their long–distance migration is naturally limited; the longest recorded distance for a single flight of *Monochamus sartor* (Fabricius, 1787) on a flight mill was 3.1 km^24^. Furthermore, the development of *M. sartor* immature stages lasts at least one year, and therefore, their populations are not a subject of such rapid demographic fluctuations such as those known in bark beetles. Moreover, several *Monochamus* species were successfully used in recent phylogeographic studies^25–29^, which pointed to the significant role of glacial events in the shaping of a genetic structure in some *Monochamus* species^26,27^.

The main aim of this paper was to describe the genetic structure and morphological variation of the sawyer beetle *M. sartor* within the vast part of a natural distribution range of its host tree, the Norway spruce. This will make it possible to investigate whether the past migration of the host in Quaternary has affected the genetic and morphological structure of its associated herbivore species.

So far, genetic variability in *M. sartor* was studied only with mitochondrial COI sequences^29^, and the results of this study suggest low but significant divergence between populations inhabiting northern and southern spruce ranges. However, mtDNA sequences provide incomplete data on the phylogeographic structure due to their maternal inheritance and susceptibility to lineage sorting, branch length stochasticity, and to introgression across taxonomic borders^18,30–32^. For this reason, we used nuclear microsatellite markers along with the geometric morphometrics approach to analyze both genetic and morphological variations between populations of *M. sartor* within the whole Palearctic range of the species—from the Western Alps, through Central and Northern Europe, to Russian Siberia and Japan. Such an approach allowed for a reliable assessment of gene flow, and potential hybridization between Alpine–Carpathian and Asian populations of the beetle. Finally, taken together with a phylogeographic data on the Norway spruce, our study aimed at shedding light on the evolutionary co-dependence between *M. sartor* and its primary host—the Norway spruce.

## Results

### Microsatellite loci variability

**Figure 1:**
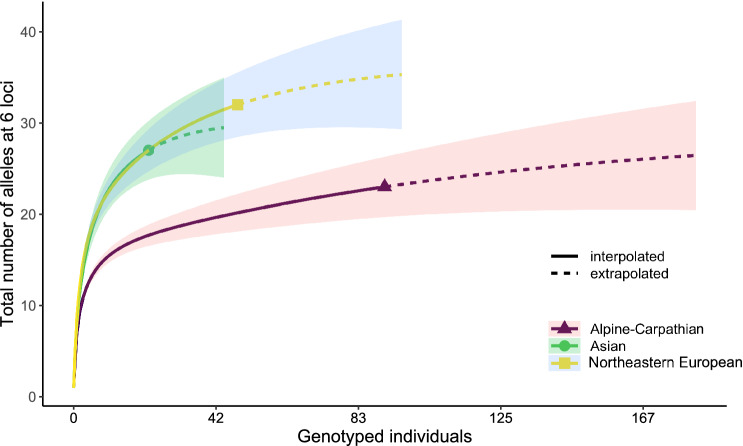
Accumulation curves of the total number of alleles at six studied microsatellite loci in three regions. Solid lines represent interpolated values, dashed lines—extrapolated values, shaded areas show associated 95% confidence intervals, as estimated by iNEXT package^33^.

In total, we subjected 210 samples to microsatellite genotyping. However, we obtained complete genotypes with no missing data for 161 of them (76.6%), and the results presented below are based on these samples only. We detected 3–12 alleles per locus and 23 to 32 alleles in total per region. Measures of microsatellite variation in the three assumed regions are given in Table 1. All genetic diversity measures were similar in Asia and northeastern Europe, and somewhat higher than in the Alpine-Carpathian region. The differences however, between the regions were not statistically significant (Table 1), despite the clear pattern revealed by the allele accumulation curves (Fig. 1).

**Table 1:** Population genetic parameters estimated for the study populations (averaged across loci).

Population	*N*	*A*	*A*_*e*_	*AR*	*H*_*o*_	*A*_*t*_
Alpine–Carpathian	91	3.83 (1.66)	2.15 (0.81)	3.39 (1.22)	0.25 (0.12)	29.24 (23.97–63.10)
Northeastern European	48	5.33 (1.20)	2.55 (0.45)	5.08 (0.84)	0.28 (0.09)	36.49 (32.81–56.82)
Asian	22	4.50 (1.15)	2.42 (0.62)	4.83 (1.11)	0.29 (0.12)	30.11 (27.50–46.24)

*N*—number of studied individuals, *A*—number of alleles, *A*_*e*_—effective number of alleles, *AR*—allelic richness (based on 44 genes), *At*—total number of alleles at six loci extrapolated with the Chao estimator, Ho—observed heterozygosity. Numbers in brackets indicate standard errors or (in the last column) 95% confidence intervals.

According to INEST results, the frequency of null alleles and genotyping failure rates were negligible for all loci. Among six studied microsatellite loci, one locus (Mon_08) showed a significant departure from the Hardy–Weinberg equilibrium in the Alpine–Carpathian population, while there were four such loci (Mon_08, Mon_30, Mon_31 and Mon_44) in the northeastern European and Asian populations, indicating that there may be a more complex population structure in these regions. We detected no significant linkage disequilibrium between any pair of loci within the three regions.

Pairwise *F*_*ST*_ between the Alpine–Carpathian and northeastern European regions was moderate and highly significant (*F*_*ST*_ = 0.119, *P* < 0.001, Table 2), as was the differentiation between the Alpine–Carpathian and Asian regions (*F*_*ST*_ = 0.181, *P* < 0.001, Table 2). In contrast, the differentiation between the northeastern European and Asian regions was relatively low (*F*_*ST*_ = 0.036) yet still highly significant (*P* < 0.001, Table 2).

**Table 2:** Matrix of the pairwise F_ST_ genetic distances based on six microsatellites among the three regions (below diagonal) and the corresponding 95% confidence intervals (above diagonal).

	Alpine–Carpathian	Northeastern European	Asian
Alpine–Carpathian		0.085–0.358	0.092–0.641
Northeastern European	0.119		0.008–0.106
Asian	0.181	0.036	

**Figure 2:**
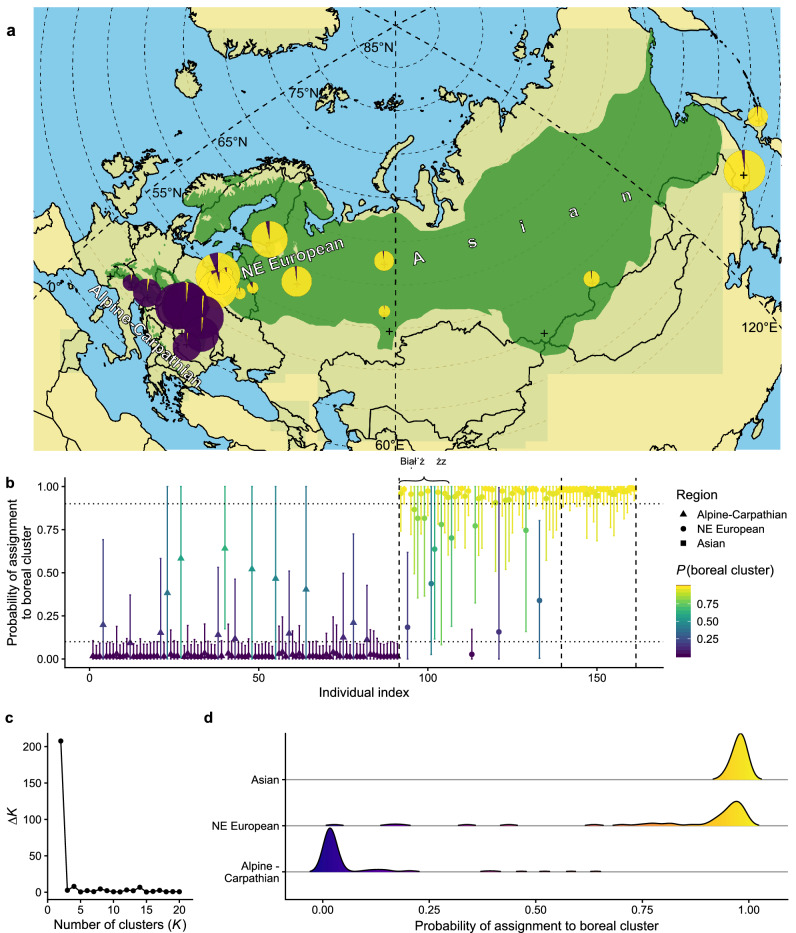
Genetic ancestry estimated for *Monochamus sartor* Fabr. samples using the software STRUCTURE: (**a)** sampling locations across the geographic range of host tree, Norway and Siberian spruce, *Picea abies* and *P. obovata* (in green), and pie charts represent genetic ancestry of each beetle populations (“ + ” are populations not included in genetic analysis but present in morphometric analysis); (**b)** individual membership probabilities of assignment to one of the two inferred clusters (Alpine–Carpathian or Eastern), with bars indicating 95% confidence intervals; (**c)** Δ*K* plot showing highest value at two (*K* = 2) as the most likely number of clusters; (**d)** distribution of assignment probabilities for the three assumed populations. Additionally, specimens from the Bialowieża Forest—potential place of the secondary contact of two *M. sartor* ranges was marked with a brace. The term ‘NE European’ is an abbreviation of the northeastern European population. The map was created using the R ver. 3.6.1 software (https://www.r-project.org/).

In the analysis with STRUCTURE, Evanno's delta K indicated that *K* = 2 was the most likely number of genetic clusters (Fig. 2c), which corresponded to two geographic lineages: one included the alpine mountain ranges of southern and central Europe (Alps and Carpathians) and the second cluster encompassed the vast boreal range (northeastern Europe and Asia) over the entire north and east part of the distribution of its host tree, *P. abies.* Such a result is consistent with the present division of the species into two subspecies, *M. s. sartor* and *M. s. urussovii* (Fig. 2d).

Most individuals sampled in these respective regions had an overwhelming majority of their ancestry assigned to the genetic cluster representing a given subspecies (Fig. 2b). From the studied individuals, 15 out of 91 from the Alpine–Carpathian region had a relatively higher probability of belonging to the eastern *M. s. urussovii* cluster (*q* > 0.1), which may be a signature of admixed ancestry. Unfortunately, because samples with a higher *q* have wide confidence intervals in all but one case, covering a range from zero to one, it is difficult to indicate them as clear evidence of admixture between lineages. Similarly, 13 out of 48 individuals from northeastern Europe had *q* < 0.9, which could be an indication of admixture from *M. s. sartor*. Again, wide confidence intervals meant that a straightforward interpretation of these values as proof of hybridization between subspecies could be misleading; however, four samples had *q* significantly smaller than one. Asian samples, all of which had *q* not significantly different from one, can undoubtedly be included in the *M. s. urussovi* cluster.

**Figure 3:**
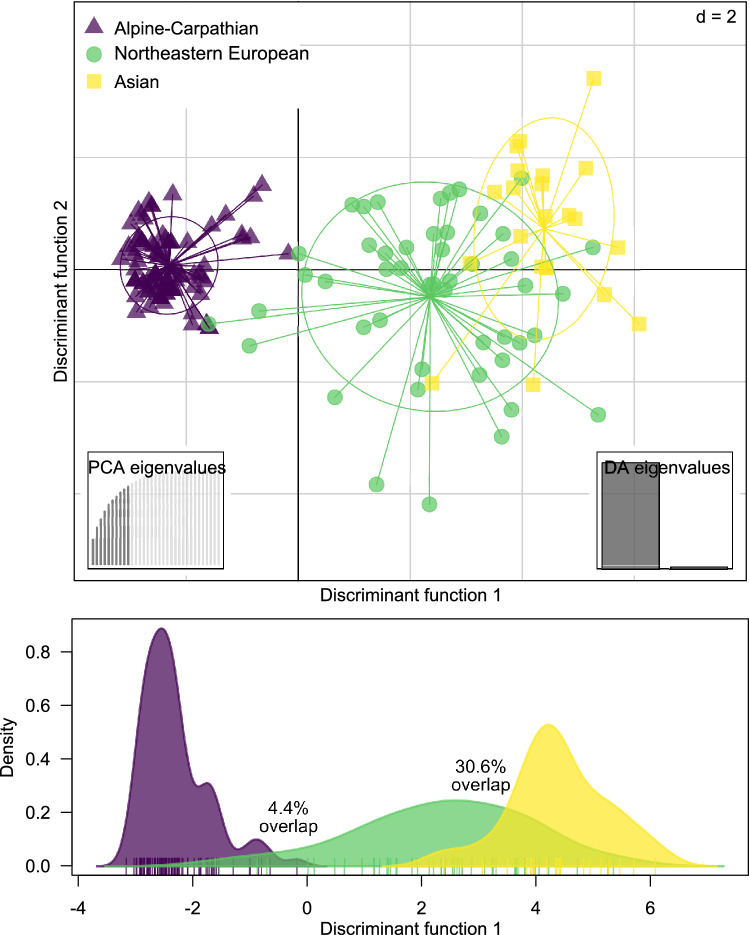
Grouping of *Monochamus sartor* Fabr. individuals across geography, using a Discriminant Analysis of Principal Components (DAPC). The density plot shows the overlap between distribution for the three assumed regions.

Similarly to STRUCTURE, the DAPC results fully supported the separation of the Alpine–Carpathian and Asian populations, although significant overlap occurred between samples from Northeastern Europe and Asia (Fig. 3). The overlap along the first discriminant axis between the Alpine–Carpathian and northeastern European populations was 4.4%, while it amounted to 30.6% between the latter and Asian populations. There was no overlap between the Alpine–Carpathian and Asian populations.

### Demographic history

**Figure 4:**
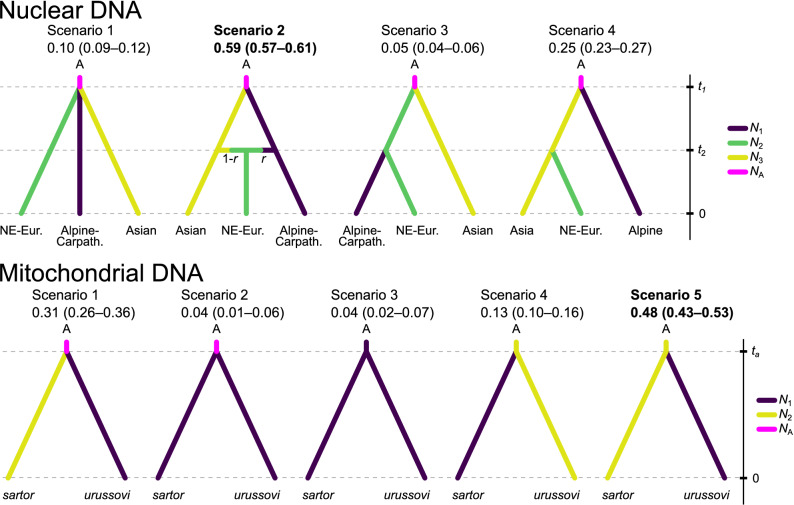
Evolutionary scenarios to describe the demographic history of *Monochamus sartor* Fabr. based on nuclear (upper panel) and mtDNA data (lower panel). The numbers below the scenario names indicate the posterior probability of each of them, with a 95% confidence interval in parentheses (the scenario with the highest probability in bold). *N*_A_—effective size of ancestral population; *N*_1_, *N*_2_, *N*_3_—effective sizes of population 1, 2, and 3; *t*_1_, *t*_2_, *t*_*a*_—divergence times, *r*—population admixture. The term ‘NE-Eur.’ is an abbreviation of the northeastern European population, and the term ‘Alpine-Carpath.’ is an abbreviation of the Alpine-Carpathian population.

Based on the nuclear microsatellite markers, the best–supported scenario describing the demographic history of *M. sartor* was the one that assumed the hybrid origin of the northeastern European population, i.e. scenario 2 (Fig. 4). Its posterior probability (PP) was 0.591 (95% CI 0.569–0.614) and was higher than the posterior probabilities of the other three scenarios (PP scenario 1 = 0.105, 95% CI 0.093–0.116; PP scenario 3 = 0.050, 95% CI 0.041–0.059; PP scenario 4 = 0.254, 95% CI 0.234–0.274).

Parameter estimates showed extensive credible intervals, likely resulting from the low information content in six genetic markers. However, the ABC approach provided the support for the scenario where Alpine-Carpathian and Asian populations represent two historical lineages, while the northeastern European population is a relatively recently admixed population, with the prevailing contribution of the Asian population (approx. 70%). According to the estimates, the Alpine-Carpathian population has a lower effective size compared to both the Asian and the northeastern European population (Table 3). Interestingly, despite a high level of uncertainty, the effective size of the ancestral population tended to be lower than the size of the current population in Asia and northeastern Europe, suggesting the possible demographic expansion of the species in the eastern part of its range.

**Table 3:** Estimates of demographic parameters for microsatellite and mtDNA data under the most likely demographic scenario.

Parameter	mean	median	mode	q025	q975
Microsatellite data (nuclear DNA)					
*N*_1_	78,000	67,700	46,500	16,600	223,000
*N*_2_	199,000	205,000	206,000	69,400	293,000
*N*_3_	167,000	167,000	167,000	46,700	286,000
*t*_2_	20,500	14,100	8330	1800	76,900
*r*	0.334	0.288	0.246	0.0229	0.889
*t*_1_	152,000	146,000	97,500	31,900	289,000
*N*_A_	96,300	73,500	491	2610	280,000
COI sequences (mtDNA)					
*N*_1_	221,000	228,000	290,000	110,000	297,000
*N*_2_ = *N*_A_	65,200	53,800	36,600	15,200	196,000
*t*_a_	53,400	41,000	25,000	9730	182,000
*μ*	5.74 × 10^–8^	5.49 × 10^–8^	4.62 × 10^–8^	2.85 × 10^–8^	9.5 × 10^–8^

In the case of mtDNA among tested scenarios (Fig. 4), Scenario 5 (in which *M. s. sartor* had the same *N*_*e*_ as ancestral population) appeared to have the highest credibility with the posterior probability of 0.48 (95% CI 0.43, 0.53). The second-best scenario was Scenario 1 (in which *N*_*e*_ of the ancestral population differed both from *M. sartor* and *M. s. urussovi*), with the posterior probability of 0.31 (95% CI 0.26, 0.36). Under the most likely scenario, the current Asian population has an effective size of 228,000 and is significantly larger than the ancestral population. On the other hand, the Alpine-Carpathian population is characterized by the effective size of 53,800 and reflects no signs of population size change since its divergence. Under the assumed mutation rate, the divergence event took place 41,000 generations ago.

### Morphological variation

Hind (metathoracic) wings were found to be significantly larger in *M. sartor* females than in males (ANOVA: F_1, 310_ = 4.79, *P* < 0.05).There was no significant difference in wing size between populations (ANOVA: F_2, 310_ = 2.18, *P* > 0.05) and the interaction between population and sex was also non–significant (ANOVA: F_2, 310_ = 1.78, *P* > 0.1).

**Figure 5:**
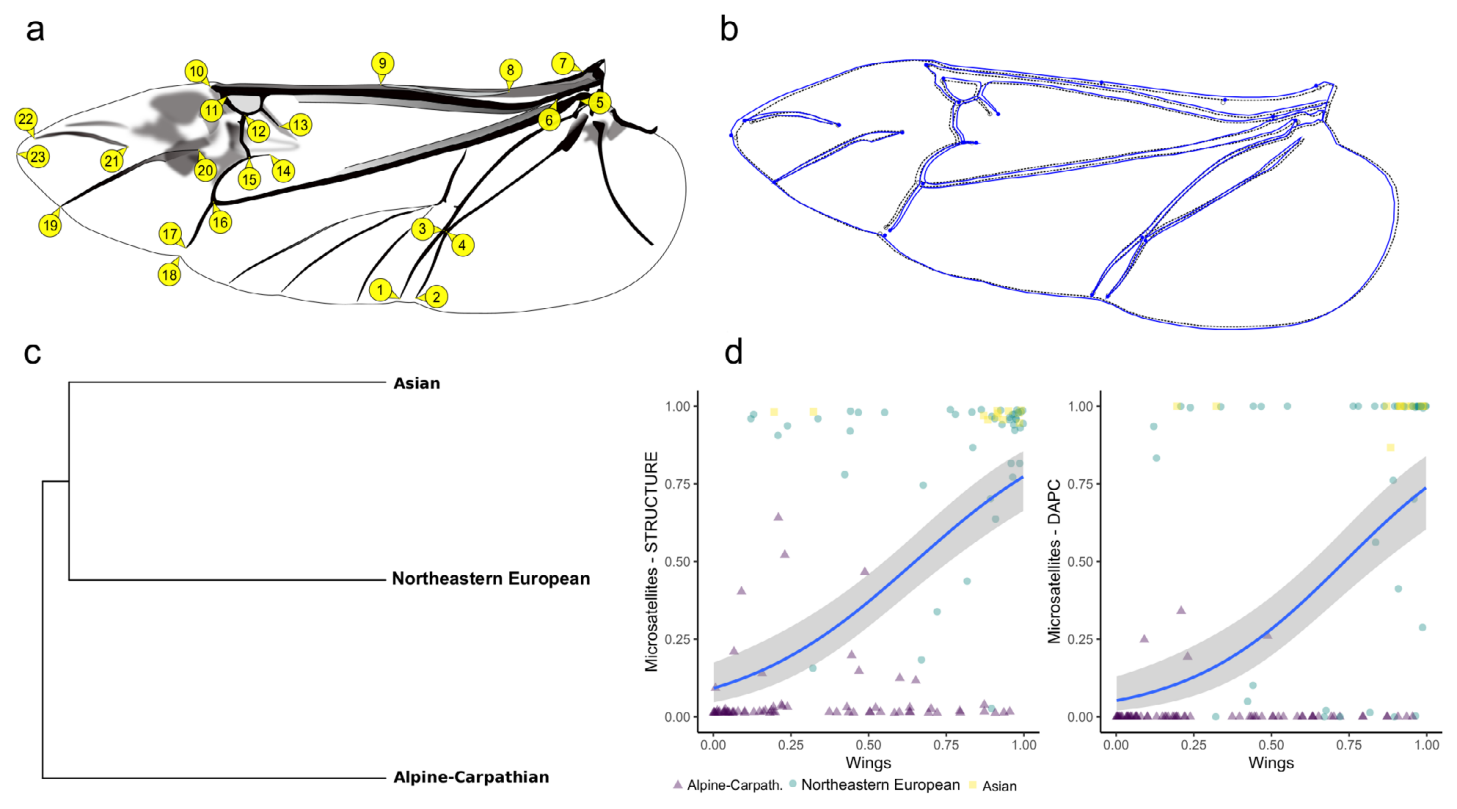
**a)** The scheme of landmark positions on the hind wing of *Monochamus sartor*; (**b)** Twice–enlarged shape differences between the averaged hind wing of *M. sartor* from Alpine–Carpathian population (black dotted line) and from the eastern range (blue line); (**c)** The UPGMA similarity tree of *M. sartor* populations based on hind wing measurements; (**d)** The relation between probabilities of assignment to the eastern cluster based on genetic analysis (y-axis) and morphological analysis of hind wing morphology (x-axis).

The crossed MANCOVA of hind wing shape with population, sex, and wing dimensions as covariates failed to detect any significant interactions between factors; therefore, we used the MANCOVA model with main effects only. The analysis revealed that the hind wing shape of *M. sartor* differs significantly between populations (Wilks' Λ = 0.35, F_40, 584_ = 10.07, *P* < 0.0001) and sexes (Wilks' Λ = 0.80, F_20, 292_ = 3.55, *P* < 0.0001). The effect of the hind wing size on wing shape was also significant (Wilks' Λ = 0.51, F_20, 292_ = 13.78, *P* < 0.0001). The highest morphological difference in wing shape was observed between Alpine–Carpathian and Asian populations (Fig. 5c), whereas the northeastern European population was more similar to the Asian population than to the Alpine–Carpathian population (Fig. 5c). The highest Mahalanobis distance (MD^2^ = 4.6) was found between the Alpine–Carpathian and Asian populations. The slightly smaller difference was observed between the Alpine–Carpathian and northeastern European populations (MD^2^ = 4.24). The northeastern European population was more similar to the Asian population (MD^2^ = 3.72, Fig. 5c) than to the Alpine–Carpathian (MD^2^ = 4.24) population. The differences in hind wing shape between populations of *M. sartor* from the Alpine-Carpathian and eastern range were rather small and difficult to observe by eye; however, they can be found in particular in wing veins located near the wing folding line (Fig. 5b).

The probability of assignment to the eastern cluster based on hind wing venation showed a good agreement with *q*-probabilities estimated with microsatellite data, as evidenced by GLM models (Fig. 5d, χ^2^(1) = 37.36 *P* < 0.001, McFadden pseudo-*R*^2^ = 0.26 for model with STRUCTURE *q-* value as a dependent variable; χ^2^(1) = 40.91, *P* < 0.001, McFadden pseudo-*R*^2^ = 0.27 for model with DAPC *q*-value as a dependent variable; note that interpretation of McFadden pseudo-*R*^2^ differs from interpretation of regular *R*^2^ from linear model, and 0.2–0.4 indicate excellent model fit^34^).

## Discussion

**Figure 6:**
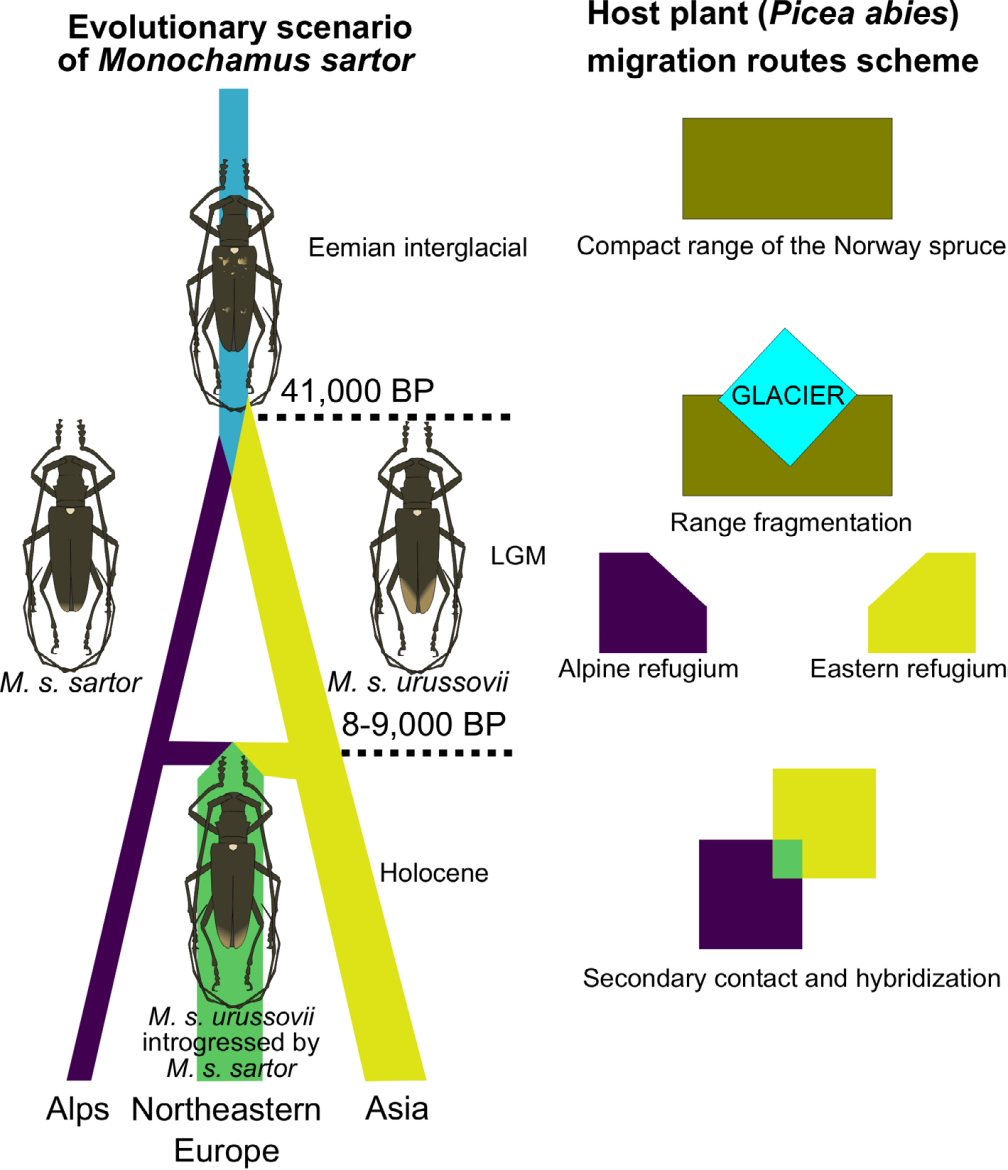
The evolutionary scenario of *Monochamus sartor* Fabr. inferred using approximate Bayesian computation (ABC) and the scheme of the Norway spruce (*Picea abies* (L.) Karst.) range fluctuations. The drawings were created using the Inscape ver. 0.92 software (https://inkscape.org/).

Extensive sampling and the combination of both molecular and morphological markers have allowed us to describe the clear geographic structure of *M. sartor* in the Palearctic realm, encompassing two main clusters: southern (Alpine–Carpathian) and eastern (northeastern Europe + Asia). However, the potential hybridization zone was found in northeastern Europe. The geographic variation of the species appeared to correspond well to the genetic patterns found in its host—the Norway spruce^11,12,14^. Moreover, our results illustrate that there is an ongoing gene flow between the southern and eastern ranges, or it has occurred in the past. An analysis of the demographic history of *M. sartor* provided the best support for the scenario involving the hybrid origin of the northeastern European population. Furthermore, our results showed that two important evolutionary events of *M. sartor*: its divergence into two lineages and its secondary hybridization (Fig. 6)—correspond with the historical demographic events connected with its host plant.

The status of the regional populations of *M. sartor* has been the subject of debates for many years^29,33–37^. Several authors pointed to the significant morphological differences between adult individuals from southern Europe (from the Alps and the Carpathians) and those from northeastern Europe and Asia^36,37^. The main differences are in the structure of dorsal setation on the apical third of elytra, which is denser in specimens from northeastern Europe and Asia^36^. However, according to the first quantitative analysis of morphological variation in *M. sartor*, specimens from northeastern Poland were found to exhibit intermediate traits between those from southern Europe and Russian Siberia, which led the authors to the conclusion that they might be hybrids formed after the secondary contact of their host plant in the Holocene^37^. A recent study based on the analysis of COI mtDNA sequences indicated that *M. sartor* populations from southern Europe and those from northeastern Europe and Asia belong to distinct mitochondrial haplogroups^29^. However, the level of divergence between them was found to be weak, which pointed authors to the suggestion that they split into two subspecies very recently, possibly at the end of the Pleistocene glaciations^29^. Nevertheless, in the previous study, limited genetic data did not allow for the verification of the hypothesis about the hybrid origin of the northeastern European population or to confirm the genetic flow between populations. Hence, more research involving nuclear DNA markers were strongly suggested^29^.

Here, based on nuclear DNA markers and comprehensive morphometric analysis, we provided strong evidence on the existence of two geographic clusters within the native Palaearctic range of *M. sartor*. The results of all conducted analyzes, including STRUCTURE, DAPC, and morphometry, fully supported the separation of the southern (Alpine–Carpathian) and eastern range of the species. Moreover, the confirmed existence of gene flow between Alpine–Carpathian and eastern cluster suggests that those subgroups should not be considered separate species, at least with regard to the generally accepted biological definition of the species. Nevertheless, the existence of a clear genetic divergence confirmed by both mitochondrial and nuclear DNA markers, external morphology, and some aspects of ecology, supports a recent change of the taxonomic status^36^ where two subspecies: *M. s. sartor* inhabiting the Alpine–Carpathian range, and *M. s. urussovii* associated with the eastern range, were distinguished.

The analysis of demographic history illustrates that *M. sartor* has most likely diverged into two lineages relatively recently, about 41,000 generations ago (Fig. 6). Given that a significant part of the population of *M. sartor* completes the development cycle in one year, even in a harsh alpine climate^24^, it can be presumed that the divergence event took place about 41,000 years ago in the Pleniglacial. During this time, its host—the Norway spruce, was experiencing significant range retreat and fragmentation into small and distant areas in Southern Europe and one large area encompassing the northeastern Europe and Palaearctic Asia^38,39^. Therefore, it is highly likely that the Norway spruce range fragmentation in the Pleniglacial has resulted in the subdivision of the initial *M. sartor* population, which consequently led to the allopatric speciation and formation of the eastern and southern subspecies. Our results shed new light also on the enigmatic population from northeastern Europe, which was found to have intermediate morphological traits between eastern and southern populations^37^. According to the most likely evolutionary scenario, the Alpine–Carpathian and Asian populations of *M. sartor* represent two historical lineages, while the northeastern European population is a relatively recently admixed population with the prevailing contribution of the Asian population (Fig. 6). Due to low information content in 6 genetic markers, the precise estimation of the time of secondary contact of two *M. sartor* lineages is challenging, however, according to the median value, it might have taken place about 8,500–8,000 years ago in the Boreal chronozone of the Holocene. According to palynological data, the Norway spruce from southern refugia started the post-glacial recolonization of present-day Polish territory around 10,000–9,000 years ago^11,40^, and reached the northern limit of the southern range about 5,000–4,000 years ago^12,40^. It is believed that the colonization of northeastern Poland by the spruce from the Russian refugium only began during this time^12^. Nevertheless, some recent data suggest that the Norway spruce could have been present in northeastern Poland markedly earlier—even during the Early Holocene about 9,500–9,000 years ago^13^. Moreover, palynological data indicates that small and sparse spruce populations could have been present in northeastern Poland long before the main expansion wave^40^. Hence, it can be speculated that *M. sartor* could have used scattered populations of Norway spruce or other conifers (secondary hosts) as stepping stones during the post-glacial recolonization, which can explain why the secondary contact of the beetle’s populations could have happened earlier than the secondary contact of the Norway spruce.

Both genetic and morphological data showed significantly lower divergence and a larger overlap between samples of *M. sartor* from northeastern Europe and Asia than between samples from northeastern European and the Alpine–Carpathian population, despite the geographic distance between northeastern Europe and Asia being markedly larger than the geographic distance between northeastern Europe and Alps or the Carpathians (Fig. 2a). These results suggest that similarly to the host tree, *M. sartor* recolonized northeastern Europe from the single refugium located in the Russian plains. Furthermore, we found that all of the genetic diversity measures which were used showed a clear tendency to be lower (although differences were not statistically significant) in the Alpine–Carpathian region rather than in the boreal range of the species (northeastern Europe and Asia), which suggests that recolonization of this area took place at high population densities; hence the population of *M. sartor* from the eastern range has no signs of a significant bottleneck effect. The same conclusion was found for its host—the Norway spruce, where genetic diversity of the northern (boreal) range was markedly higher than in the southern Alpine–Carpathian region, leading to the conclusion that the Russian refugium had once occupied a large area^11,12^. Our data suggests higher levels of gene flow from southern populations to the northeast than in the opposite direction, which is consistent with the postglacial migration patterns of the Norway spruce, where the southern recolonization wave was found to be markedly more expansive^11,12,40^.

Replacing the simple linear measurements with geometric morphometrics which involves coordinated landmarks placed on homologous morphological structures, has provided new perspectives in studies on taxonomy, paleontology as well as developmental and evolutionary biology^41,42^. In the presented study, we found a good agreement between morphometric and molecular methods in distinguishing *M. sartor* subspecies. Similar agreement was also found in the case of other insects^43,44^. Although microsatellites are more powerful than morphometry, they are not always developed for a particular species. Moreover, their cost is considerably higher in comparison to morphometry. We have started our study with measuring a smaller sample of *M. sartor* and found an interesting pattern of shape variation^37^. This study allowed us to confirm the morphological data about the differences between populations and to describe them more precisely. Morphological analysis can be considered as an easy to use and inexpensive exploratory tool allowing for the detection of interesting patterns. It should be noted, however, that not all differences in shape need to be genetically determined. In many species, including *M. sartor*, there is significant allometry—relationship between shape and size. In this situation, appropriate statistical tools should be used to analyze various sources of the shape variation.

Although the six genetic markers provided enough data to disentangle subspecies, it appeared to be insufficient for the genetic assignment at the individual level, at least for some individuals. For this reason, we were not able to estimate precisely the rate of hybridization between subspecies. The limited power of markers also had an impact on the quality of estimates of demographic parameters derived from the ABC approach, despite relatively robust conclusions regarding the scenario of recent species evolution.

A large effective population size is difficult to estimate precisely because it is inversely proportional to molecular signatures of genetic drift. In the case of coalescent–based methods, the same applies to time which is measured in terms of effective size. In addition, the ABC approach requires the assumption of a mutation model (including mutation rates), which may have a great impact on the estimated value of parameters. Taken together, the limited power of markers and the inherent properties of the ABC method used in this study caused poor–quality estimates of the effective size and times to the divergence or split of the study populations. Therefore, in order to better characterize the recent evolutionary scenarios, future studies should focus on high–throughput molecular methods.

One of the factors that might potentially affect the geographic structure of *M. sartor* could be the artificial planting of *P. abies* outside its native range. Such artificial spruce plantations have already occurred in some parts of the “sprucless zone” running through the Middle–Polish Plains^14^. Although *M. sartor* has limited dispersal abilities, the scattered artificial plantations of spruce can potentially serve as a stepping-stone habitat during the population spread^26^, and consequently lead to the hybridisation of the Alpine–Carpathian and northeastern European populations. Dispersal of this species might also be affected by the artificial relocation of individuals with infected wood. It is widely known that premature stages of wood-boring beetles (e.g. bark beetles and longhorn beetles) can survive a long time in fresh timber or even in manufactured wood, and start to spread rapidly after reaching new optimal habitats^45^. Nevertheless, our data showed that both divergence and secondary contact of *M. sartor* lineages took place well before the forest management began.

Although our study provided new and interesting insights into the evolutionary co-dependence between the Norway spruce and its herbivore—*M. sartor*, there are still some questions that need to be addressed in the future. Firstly, the application of more sophisticated high–throughput molecular methods will allow for a more reliable estimation of both divergence time and gene flow between the populations. Furthermore, many studies on closely related species highlighted the role of altitude in *Monochamus* radiation^28^, however, our sample cover did not allow for the testing of the same hypothesis in *M. sartor*. Therefore, more samples from the two sides of Alps and Ural chains should be analyzed in the future. It was shown that both geographic barriers and glacial events shaped the complex geographic structure of many tree-feeding longhorn beetles in the Far East, especially in the Japanese Islands^26,46–48^^.^ More samples from this region will allow for the investigation on how the aforementioned factors have affected the genetic structure of *M. sartor* at the eastern border if its distribution. It is also still not fully understood whether the observed morphological difference (e.g. structure of dorsal setation) between *M. sartor* populations have genetic or rather environmental origin. Therefore, common garden experiments with an artificial hybridization of *M. s. sartor* and *M. s. urussovii* might provide interesting insights into the origin of the observed morphological patterns, mechanism of introgression, and the fitness of the hybrids. Lastly, the current climate change and related spruce decline observed in many regions of Europe provides interesting opportunity to study the genetic dynamic of *M. sartor* metapopulation under the rapid climatic and ecological change.

## Materials and methods

### Target species

*M. sartor* is a medium size (up to 34 mm body length^36^) sawyer beetle. Larval development usually takes place in the wood of weakened or recently dead spruces *P. abies* which is the species’ primary host plant, and rarely in several species from the genus *Abies* Mill, *Pinus* L., and *Larix* Miller^29,49^. Based on published data from eastern Russia, occasionally this species may also develop in some tree species from the genus *Betula* L., but the record needs further confirmation^50^. The range of the species coincides roughly with the spruce range in Eurasia (Fig. 2a—map), and includes the mountain ranges of central and southern Europe (the Alps and Carpathians), northeastern Europe, the Baltic countries, Fennoscandia, most of Siberia, and the Russian Far East up to the northern (forested) areas of Mongolia, China, the Korean Peninsula, and Japan (Hokkaido), extending north beyond the Arctic Circle to the northern border of the taiga biome^35,37,50,51^.

The taxonomy of *M. sartor* is not fully understood. A recent molecular study based on the COI marker, morphological comparisons, and the levels of *Wolbachia* diversity indicated that the species could be split into two genetically distinct lineages: the first one was found to inhabit the Alps and Carpathians, and second—northeastern Europe and Asia^29^. Such results supported the recent taxonomic study, where *M. sartor* was divided into two subspecies by combining two taxa previously considered separate species: *M. s. sartor* (Fabricius, 1787) (distributed among the mountains of Central and Southern Europe) and *M. s. urussovii* (J.G. Fischer von Waldheim, 1805) (distributed throughout eastern and northern Europe, Russia, Mongolia, China, the Korean Peninsula, and Japan)^36^. Recent morphological studies suggested, however, a possible gene flow between *M. sartor* subspecies during the potential secondary contact in the Holocene, as individuals from northeastern Poland had intermediate morphological traits^37^.

### Sample collection and identification

Our sampling covered both *M. sartor* subspecies over roughly the entire species range, from the Alps in the West to Japan in the East (Fig. 2a). We grouped samples in three geographic subsamples (populations), according to their geographic location: (1) Alpine–Carpathian (the Alps and Carpathians), (2) northeastern European (the European part of the boreal range, including northeastern Poland, the Baltic Countries, Scandinavia, and the European part of Russia) and (3) Asian (from the Urals to Japan). The term “eastern range”, used in this paper, refers to the whole boreal range of *M. sartor* including both the northeaster European and Asian populations, while the term “southern” range refers to the Alpine-Carpathian populations of *M. sartor*.

Most of the samples were captured between 2016 and 2018 using pheromone traps or sampled manually from wood stocks of *P. abies*. Specimens were identified in the laboratory using a published key^36^. In total, 352 specimens originated from various regions (Fig. 2a, Supplementary materials 1) within the major part of the species distribution range were processed. For the morphological analysis, we used 316 specimens (161♂, 155♀), whereas 210 specimens (72♂, 54♀, 36—unidentified sex) were used in the genetic analysis (see Supplementary Material 1 for details). Due to difficulties with fresh material collection in some regions, we used dry materials kindly provided upon request by entomologists (Supplementary material 1).

### Molecular genetic analysis

DNA was extracted from insect legs using the Insect Easy DNA Kit (EZNA) (Omega Bio–Tek) following the manufacturer’s protocol.

We used microsatellite loci originally developed for *Monochamus galloprovincialis* (Olivier 1795)^52^. Out of 12 primer pairs published for the congeneric species, we obtained the successful amplification of six loci in our *M. sartor* samples (Mon_08, Mon_17, Mon_30, Mon_31 Mon_36 Mon_44). All loci were amplified in a single multiplex reaction using the Multiplex PCR Kit reagent kit (QIAGEN, Inc.) according to the protocol recommended by the manufacturer. Details on PCR conditions, fragment separation and genotyping are described Supplementary Material 2.

Since the genotyped material included both fresh insects preserved in ethanol and dry specimens from entomological collections, the individual samples differed in the success of the amplification, likely due to DNA degradation. In cases when one or more loci failed to amplify, we repeated the multiplex PCR for said particular sample up to three times. Finally, samples with missing data were excluded. Additionally, individuals that were identified as potential hybrids between subspecies (see the following paragraphs) were genotyped twice.

We assessed the potential presence of null alleles and genotyping failure rate using INEST ver. 2.1^53^, departure from HWE using the exact test^54^ in ‘pegas’ ver. 0.12^55^, linkage disequilibrium using the standardized index of association, $\bar{r}_{\text{d}}$^56^ in ‘poppr’ ver. 2.8.3^57^. Multiple testing was corrected using the *q*–value method (*q* < 0.05) with ‘qvalue’ ver. 2.14.1^58^.

For comparison of genetic variation, we calculated the number of alleles (*A*), the effective number of alleles (*A*_*e*_), allelic richness (*AR*) and observed heterozygosity (*H*_*o*_), using ‘hierfstat’ ver. 0.4.22^59^. In addition, since *AR* may underestimate the genetic diversity in the presence of rare alleles, we attempted to extrapolate the allele accumulation curves of the less complete samples instead of rarefying the more complete samples to predict the real diversity considering the expected number of alleles undetected by the sampling effort. The analysis was performed in ‘iNEXT’ ver. 2.0.19^60,61^.

To measure the extent of divergence between the three regions, pair–wise *F*_*ST*_ was estimated using ‘hierfstat’ ver. 0.4.22^59^, with significance tested using 100,000 permutations.

To infer the population ancestry and potential gene flow between regions, we used STRUCTURE ver. 2.3.4^62^. In the analysis, we used no prior information about sample origin, assumed correlated allele frequencies, and allowed for population admixture. To determine the most likely number of clusters (*K*), we used the Δ*K* aproach^62^.

Since STRUCTURE relies on a specific population genetic model with restrictive explicit assumptions that may not be met in the studied beetles (i.e. populations at Hardy–Weinberg equilibrium and linkage equilibrium between loci), we also investigated population subdivision using discriminant analysis of principal components (DAPC)^63^ with ‘adegenet’ ver. 2.1.1 package^64^. Two–dimensional scatter plots were constructed to visualize the spread of the first two discriminant functions, both between and within assumed regions. Since most of the variability between regions was distributed along the first discriminant axis, we calculated the overlap between density distributions as a measure of the distinctness of the regions. The calculation was performed using ‘overlapping’ ver. 1.5.4 in R ver. 3.6.1^65^.

### Coalescent analysis of demography

To test different hypotheses of *M. sartor* demography, we performed an approximate Bayesian computation (ABC) analysis implemented in DIYABC ver. 2.1.0^66^. We performed two groups of analyses, (1) using multilocus microsatellite genotypes, and (2) based on previously published^29^ cytochrome oxidase (*cox*I) sequences of mtDNA (MF327393–MF327421 and MF371175–MF371201). In both cases, we tested several scenarios. The choice of scenarios was based on the current knowledge of the evolutionary history of the Norway spruce, as well as the properties of markers (e.g. no recombination in mtDNA). The analyses are described in full detail in the Supplementary Material 2.

In the case of microsatellite data, we considered four evolutionary scenarios (Fig. 4). The following three recent groups (time = 0) were assumed to have originated from an unknown ancestor population (A): Alpine–Carpathian, northeastern European, and Asian populations. In the case of mtDNA sequence data, five competing scenarios with two sampled populations (the Alpine-Carpathian and the Asian subspecies range) were tested (Fig. 4). In order to perform the analysis, 1,000,000 data trials were simulated, assuming that each scenario had an equal prior probability. The resulting reference table was then used to estimate the posterior probability for each scenario based on the logistic regression approach^66^.

### Geometric morphometrics analysis of hind wing venation

Hind wings of 316 specimens of *M. sartor* originated from three geographic regions (see Materials and Methods—Sample collection and identification) were carefully dissected using precise tweezers, cleaned in alcohol, straightened with a small brush, and dry mounted between two microscope slides. Subsequently, high–quality images of each preparation were made by using a 10 megapixel digital camera equipped with a Ricoh 25 mm lens. The camera was mounted horizontally on a column stand. For all wing images, 23 landmarks (Fig. 5a) were determined manually using IdentiFly (v. 1.2.) software^67^.

The coordinates of the landmarks were aligned using generalized orthogonal least–squares procedures^68^. Wing shape was described by 20 uncorrelated and size–independent principal components, selected using the scree test^69^. Wing size was described by a centroid size. Mixed-design analysis of variance (ANOVA) models were used to analyze the differences in wing size between populations and sexes, whereas mixed-design multivariate analysis of covariance (MANCOVA) models were used to analyze differences in hind wing shape in Statistica v. 13 software (TIBCO Software Inc.)^70^. The Mahalanobis distance was used as a measure of the morphological divergence among populations and to construct the tree of morphological similarity between populations by using the unweighted pair-group method with arithmetic mean (UPGMA) method in ‘phangorn’ ver. 2.5.5.^71^ package in R^72^. We also carried out discriminant analysis between both the eastern and southern ranges of *M. sartor* to obtain the posterior probabilities of the assignment of each specimen to one of two clusters.

We tested the agreement between probabilities of individual assignments to subspecies based on multilocus microsatellite genotypes and wing venation by fitting the fractional response regression model that is suitable for rates, proportions, and probabilities used as dependent variables. The model was specified as a generalized linear model (GLM) with the quasibinomial error structure and the logit link function. For genetic data, treated as dependent variables, we used two measures of the probability of subspecies assignment, STRUCTURE *q*–values and DAPC *q*–values, in both cases obtained for *K* = 2 without any prior information on sample location.

## Supplementary information

Supplementary information 1

Supplementary information 2
