# Supplementary material for: Climatic oscillations in Quaternary have shaped the co-evolutionary patterns between the Norway spruce and its host-associated herbivore
Source: Sci Rep. 2020 Oct 5;10:16524. doi: 10.1038/s41598-020-73272-0 (PMC7536422; doi:10.1038/s41598-020-73272-0)
Supplement: Supplementary file 2 — Supplementary information 2 [file 41598_2020_73272_MOESM2_ESM.doc]

**Supplementary Material 2 for:**

**Climatic oscillations in Quaternary have shaped the co-evolutionary patterns between the Norway spruce and its host-associated herbivore**

^1*^Jakub Goczał, ^2*^Andrzej Oleksa, ^3^Robert Rossa, ^4^Igor Chybicki, ^5^Katarzyna Meyza, ^6^Radosław Plewa, ^7^Matti Landvik, ^8^Mauro Gobbi, ^9^Gernot Hoch, ^10^Vytautas Tamutis, ^11^Maksims Balalaikins, ^12^Dmitry Telnov,, ^13^Maria-Magdalena Dascălu, ^14^Adam Tofilski

# ^1,3^Department of Forest Ecosystems Protection, Faculty of Forestry, University of Agriculture in Krakow, 29 Listopada 46, 31-425 Kraków, Poland, e-mail: JG - j.goczal@wp.pl, RR - rlrossa@cyf-kr.edu.pl

# ^2,4,5^Department of Genetics, Faculty of Biological Sciences, Kazimierz Wielki University, Powstańców Wielkopolskich 10, 85-090 Bydgoszcz, Poland, e-mail: AO - olek@ukw.edu.pl, IC - igorchy@ukw.edu.pl, KM - [kasiakow@ukw.edu.pl](mailto:kasiakow@ukw.edu.pl)

^6^Department of Forest Protection, Forest Research Institute, Sękocin Stary, Braci Leśnej 3, 05-090 Raszyn, Poland, e-mail: r.plewa@ibles.waw.pl

^7^Tainionkoskentie 26 a. 2, 55100, Imatra, Finland, e-mail: [matti.landvik@gmail.com](mailto:matti.landvik@gmail.com)

^8^Section of Invertebrate Zoology and Hydrobiology, MUSE-Science Museum, Corso del Lavoro e della Scienza 3, 38122 Trento, Italy, e-mail: mauro.gobbi@muse.it

^9^BFW – Austrian Research Centre for Forests, Seckendorff-Gudent-Weg 8, 1131 Vienna, Austria, email: gernot.hoch@bfw.gv.at

^10^Kaunas Botanical Garden, Vytautas Magnus University, Ž.E. Žilibero str. 6, LT-46324, Kaunas,Lithuania, e-mail: dromius@yahoo.com

^11^Institute of Life Sciences and Technology, Daugavpils University, Vienibas 13, Daugavpils, LV-5400, Latvia, e-mail: maksims.balalaikins@biology.lv

^12^Department of Life Sciences, Natural History Museum, SW7 5BD, London, United Kingdom, e-mail: anthicus@gmail.com

^12^Institute of Biology, University of Latvia, Miera iela 3, LV–2169, Salaspils, Latvia, e-mail: anthicus@gmail.com

^13^Research Group in Invertebrate Diversity and Phylogenetics, Faculty of Biology, Alexandru Ioan Cuza University, Bd. Carol I, nr. 11, 700506, Iasi, Romania, dascalumm@yahoo.com

^14^Department of Zoology and Animal Welfare, University of Agriculture in Krakow, Adama Mickiewicza 24/28, 30-059 Kraków, Poland, e-mail: rotofils@cyf-kr.edu.pl

*Corresponding authors: ^1^Jakub Goczał, ^2^Andrzej Oleksa, e-mail: JG - j.goczal@wp.pl, AO - olek@ukw.edu.pl

# Microsatellite amplification

Six microsatellite loci (Mon_08, Mon_17, Mon_30, Mon_31 Mon_36 Mon_44) were amplified in a single multiplex reaction using the Multiplex PCR Kit reagent kit (QIAGEN, Inc.) according to the protocol recommended by the manufacturer (5 μL of 2 × QIAGEN Multiplex Master Mix, 50 nM of each primer of primer mix and 10 ng of template DNA and ddH20 up to the total volume of 10 μL). The thermal PCR conditions were as follows: an initial incubation at 95°C for 15 min; 9 touchdown cycles: 94°C for 30 seconds, 60°C (–0.5 ° C per cycle) for 1 min. 30 s and 72 ° C for 1 min; 24 cycles of 94 ° C for 30 s, 55 ° C for 1 min. 30 s and 72 ° C for 1 min; final elongation at 72 ° C for 10 min. For amplification, we used PTC200 thermal cycler (MJ Research). Separation of the amplified fragments was performed on an ABI PRISM 3130xl automated sequencer (Applied Biosystems) using a LIZ 600 size marker (Applied Biosystems). The electropherograms were interpreted using the GeneMarker ver. 2.6.3 (Softgenetics).

# Microsatellite loci variability

We assessed the potential presence of null alleles and genotyping failure rate using INEST ver. 2.1^1^, with analysis carried out separately for three assumed regions. We tested departure from Hardy–Weinberg equilibrium (HWE) using the exact test based on Monte Carlo permutations of alleles ^2^ in ‘pegas’ ver. 0.12^3^ with 10,000 permutations. We tested linkage disequilibrium between alleles of each pair within each of the three geographic regions using the standardized index of association,
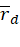
 ^4^, which was calculated using ‘poppr’ ver. 2.8.3.^5^. Multiple testing was corrected using the *q*–value method (*q* < 0.05) with ‘qvalue’ ver. 2.14.1^6^.

For comparison of genetic variation in three regions, we calculated the number of alleles (*A*), the effective number of alleles (*A_e_*), allelic richness (*AR*) and observed heterozygosity (*H_o_*), using ‘hierfstat’ ver. 0.4.22^7^. In addition, since *AR* may underestimate the genetic diversity in the presence of rare alleles, we applied to extrapolate the allele accumulation curves of the less complete samples instead of rarefying the more complete samples to predict the real diversity considering the expected number of alleles undetected by the sampling effort. The analysis was performed in ‘iNEXT’ ver. 2.0.19^8,9^.

To measure the extent of divergence between the three regions, pair–wise *F_ST_* was estimated using ‘hierfstat’ ver. 0.4.22^7^, with significance tested using 100,000 permutations.

# STRUCTURE analysis

To infer the population ancestry and potential gene flow between regions, we used STRUCTURE ver. 2.3.4^10^. In the analysis, we used no prior information about sample origin, assumed correlated allele frequencies and allowed for population admixture (default options). It has been demonstrated that such assumptions work best in studies of closely related populations with some amount of gene flow^11^.

To determine the most likely number of subpopulations (*K*) we used Δ*K* statistics^12^ computed based on ten runs (each involving a burn–in period of 250,000 and with 500,000 Markov chain Monte Carlo steps) for K values in the range from 1 to 21 using the ‘pophelper’ R package^13^. Finally, we made an elongated run for the optimal *K*, which included 500,000 burn–in and 1 million Markov chain steps.

# DAPC analysis

Since STRUCTURE relies on a specific population genetic model with restrictive explicit assumptions that may not be met in the studied beetles (i.e. populations at Hardy–Weinberg equilibrium and linkage equilibrium between loci), we investigated population subdivision also using discriminant analysis of principal components (DAPC)^14^ with ‘adegenet’ ver. 2.1.1 package^15^ – a model–free multivariate approach. This method relies on data transformation using principal component analysis (PCA) as a prior step to obtain uncorrelated variables for discriminant analysis. As a result, the method provides the best discrimination of individuals into pre–defined groups (in our study, beetles from three regions). Retaining too many PCs for discriminant analysis may lead to overfitting the discriminant functions, which could virtually discriminate any set of clusters. Therefore, ‘adegenet’ offers an optimisation procedure to assess the optimal number of PCs to retain, which is based on the calculation of the α–score, i.e. a statistics that measures the difference between the proportion of successful reassignment of the analysis (observed discrimination) and values obtained using random groups (random discrimination). Following the recommendation of ‘adegenet’ developers, we used the highest mean alpha across all simulations as an indication of the optimum number of PCs to retain. In the discriminant analysis, we kept only the first two eigenvalues, as they captured the vast majority of the information. Two–dimensional scatter plots were constructed to visualise the spread of the first two discriminant functions between and within assumed regions. Since most of the variability between regions was distributed along the first discriminant axis, we calculated the overlap between density distributions as a measure of the distinctness of the regions. The calculation was performed using ‘overlapping’ ver. 1.5.4 in R^16^.

# Coalescent analysis of demography

To test different hypotheses of *M. sartor* demography, we performed an approximate Bayesian computation (ABC) analysis implemented in DIYABC ver. 2.1.0^17^. We performed two groups of analyses, (1) using multilocus microsatellite genotypes, and (2) based on cytochrome oxidase (*cox*I) sequences of mtDNA published by Plewa et al. (2018) and available in GeneBank under accession numbers: MF327393–MF327421 and MF371175–MF371201. In both cases, we tested several scenarios. The choice of scenarios was based on the current knowledge of the evolutionary history of Norway spruce as well as the properties of markers (e.g. no recombination in mtDNA).

In the case of microsatellite data, we considered four evolutionary scenarios (Fig. 4). The following three recent groups (time = 0) were assumed to have originated from an unknown ancestor population (A): Alpine–Carpathian, NE European, and Asian populations. For demographic parameters, *N*_1_, *N*_2_ and *N*_3_, prior distributions were set as uniform distributions between 100 and 300,000. For the ancestral population size (*N_A_*), the prior was set as a uniform distribution between 100 and 500,000. For divergence times, *t*_1_ and *t*_2_, where *t*_2_<*t*_1_, the priors was set as uniform distribution between 1,000 and 300,000 for *t*_1_ and between 10 and 300,000 for *t*_2_. In the case of mutation models, we used the default settings. The reference table was built based on 1,000,000 random coalescent samples.

The priors described above reflected the following assumptions: (i) current populations are quite large (at least 100 individuals effectively); (ii) the time of divergence of the ancestral population into two (or three) lineages is at least 1,000 generations (ago); (iii) the time of population admixture (scenario 2), and the emergence of NE European population is less than the time of initial divergence from the ancestral population.

Assumptions for the Generalized Stepwise Mutation Model reflected a low polymorphism level found in the study populations: because the current effective population size is believed to be large, we assumed that the mutation rate must be low. Trial coalescent simulations showed that when the prior distribution for the mutation rate is assumed to include higher values (e.g. 1E–2), the observed data laid far outside the cluster of simulated data (results not shown).

We selected 28 summary statistics. One–sample statistics included the mean number of alleles, mean genetic diversity, mean variance of allele size, mean Garza–Williamson index. Two–sample summary statistics included mean number of alleles, mean genetic diversity, mean variance of allele size, pairwise *F_ST_*, and (*dμ*)^2^ distance. Also, the admixture statistics of Choisy et al. (2004) was included (NE European as the admixed population).

In the case of mtDNA sequence data, five competing scenarios with two sampled populations (the alpine and the Asian subspecies range) were tested (Fig. 4). The following demographic parameters were estimated: *N*_1_ and *N*_2_ (the effective size of sampled populations), *N_A_* (the effective size of the ancestral population) and ta (the time to divergence into two species’ ranges). We ran the analysis under the assumption of HKY85 mutation model, which appeared to be the most plausible among available mutation models, according to the results of the estimation of the genetic tree. For demographic parameters *N*_1_, *N*_2_ and *t_a_*, prior distributions were set as uniform distributions between 10 and 300,000. For the ancestral population size *N_A_*, the prior was set as a uniform distribution between 10 and 500,000. In the case of mutation models, we used the default settings. The reference table was built based on 1,000,000 random coalescent samples. We used the following one–sample summary statistics: number of haplotypes, number of segregating sites, mean pairwise differences, the variance of pairwise differences, Tajima’s *D*, private segregating sites, mean number of the rarest nucleotide at segregating sites, the variance of numbers of the rarest nucleotide at segregating sites. In addition, we used the following two–sample summary statistics: number of haplotypes, number of segregating sites, mean pairwise differences within and between samples, *F_ST_* parameter of Hudson et al. (1992).

In order to perform the analysis, 1,000,000 data were simulated, assuming that each scenario has an equal prior probability. The resulting reference table was then used to estimate the posterior probability for each scenario based on the logistic regression approach^17^.

# References

1. Chybicki, I. J. & Burczyk, J. Simultaneous estimation of null alleles and inbreeding coefficients. *J. Hered.* **100**, 106–113 (2009).

2. Guo, S. W. & Thompson, E. A. Performing the Exact Test of Hardy-Weinberg Proportion for Multiple Alleles. *Biometrics* **48** 361–372 (1992).

3. Paradis, E. Pegas: An R package for population genetics with an integrated-modular approach. *Bioinformatics* **26** 419–420 (2010).

4. Agapow, P. M. & Burt, A. Indices of multilocus linkage disequilibrium. *Mol. Ecol. Notes* **1**, 101–102 (2001).

5. Kamvar, Z. N., Tabima, J. F. & Gr̈unwald, N. J. Poppr: An R package for genetic analysis of populations with clonal, partially clonal, and/or sexual reproduction. *PeerJ* **2014**, 1–14 (2014).

6. Storey, J. D., Bass, A. J., Dabney, A. & Robinson, D. qvalue: Q-value estimation for false discovery rate control. R package version 2.14.1. http://github.com/jdstorey/qvalue. (2019).

7. Goudet, J. & Jombart, T. hierfstat: estimation and tests of hierarchical F-statistics. http://www.r-project.org, http://github.com/jgx65/hierfstat. (2015).

8. Hsieh, T. C., Ma, K. H. & Chao, A. iNEXT: iNterpolation and EXTrapolation for species diversity. R package version 2.0.19 URL: http://chao.stat.nthu.edu.tw/blog/software-download/. (2019).

9. Chao, A. *et al.* Rarefaction and extrapolation with Hill numbers: a framework for sampling and estimation in species diversity studies. *Ecol. Monogr.* **84**, 45–67 (2014).

10. Pritchard, J. K., Stephens, M. & Donnelly, P. Inference of population structure using multilocus genotype data. *Genetics* **155**, 945–959 (2000).

11. Falush, D., Stephens, M. & Pritchard, J. Inference of population structure using multilocus genotype data: linked loci and correlated allele frequencies. *Genetics* **164**, 1567–1587 (2003).

12. Evanno, G., Regnaut, S. & Goudet, J. Detecting the number of clusters of individuals using the software STRUCTURE: a simulation study. *Mol. Ecol.* **14**, 2611–2620 (2005).

13. R Core Team. R: A language and environment for statistical computing. *R Foundation for Statistical Computing, Vienna, Austria. URL https://www.R-project.org/* (2019).

14. Jombart, T. *et al.* Discriminant analysis of principal components: a new method for the analysis of genetically structured populations. *BMC Genet.* **11**, 94 (2010).

15. Jombart, T. Adegenet: A R package for the multivariate analysis of genetic markers. *Bioinformatics* **24**, 1403–1405 (2008).

16. Pastore, M. Overlapping: a R package for Estimating Overlapping in Empirical Distributions. *J. Open Source Softw.* **3**, 1023 (2018).

17. Cornuet, J.-M. *et al.* DIYABC v2.0: a software to make approximate Bayesian computation inferences about population history using single nucleotide polymorphism, DNA sequence and microsatellite data. *Bioinformatics* **30**, 1187–1189 (2014).

18. Plewa, R. R. *et al.* Morphology, genetics and Wolbachia endosymbionts support distinctiveness of Monochamus sartor sartor and M. s. urussovii (Coleoptera: Cerambycidae). *Arthropod Syst. Phylogeny* **76**, 123–135 (2018).

19. Choisy, M., Franck, P. & Cornuet, J. M. Estimating admixture proportions with microsatellites: Comparison of methods based on simulated data. *Mol. Ecol.* **13**, 955–968 (2004).

20. Hudson, R. R., Slatkin, M. & Maddison, W. P. Estimation of levels of gene flow from DNA sequence data. *Genetics* **132**, 583–589 (1992).
